# Sarcoptes infestation in two miniature pigs with zoonotic transmission – a case report

**DOI:** 10.1186/s12917-018-1420-5

**Published:** 2018-03-13

**Authors:** Alexander Grahofer, Jeanette Bannoehr, Heiko Nathues, Petra Roosje

**Affiliations:** 1 Clinic for Swine, Department of Clinical Veterinary Medicine, Vetsuisse Faculty, University of Bern, Bremgartenstrasse 109a, 3012 Bern, CH Switzerland; 2 Dermatology Department, Animal Health Trust, Lanwades Park, Kentford, Newmarket, Suffolk, Cardiff, CB8 7UU UK; 3 Division of Clinical Dermatology, Department of Clinical Veterinary Medicine, Vetsuisse Faculty, University of Bern, Bern, Länggassstrasse 128, 3012 Berne, CH Switzerland

**Keywords:** Pet pig, Zoonosis, Sarcoptic mange, Pruritus, Mite, Hypotrichosis

## Abstract

**Background:**

Scabies is a contagious skin disease rarely described in miniature pigs. To the best of the authors’ knowledge, a zoonotic transfer from infected pet pigs to humans has not been reported previously.

**Case presentation:**

This case report describes the infestation with *Sarcoptes scabiei* mites in two miniature pigs presenting with unusual clinical signs, and disease transmission to a child.

Two 7-month-old male castrated miniature pig siblings were examined. Both had developed skin lesions, one animal was presented for neurological signs and emaciation. They were housed together in an indoor- and outdoor enclosure. Dermatological examination revealed a dull, greasy coat with generalized hypotrichosis and multifocal erythema. Microscopic examination of skin scrapings, impression smears of affected skin and ear swabs revealed high numbers of Sarcoptes mites in both animals as well as bacterial overgrowth. A subcutaneous injection of ivermectin 0.3 mg/kg was administered to both animals and repeated after 2 weeks. Both miniature pigs received subcutaneous injections with butafosfan and cyanocobalamin, were washed with a 3% chlorhexidine shampoo and were fed on a well-balanced diet. Pig enclosures were cleaned. The infested child was examined by a physician and an antipruritic cream was prescribed. Both miniature pigs and the child went into clinical remission after treatment.

**Conclusion:**

Sarcoptic mange is rare or even eradicated in commercial pig farming in many countries but miniature pigs may represent a niche for *Sarcoptes scabiei* infections. This case report indicates that miniature pigs kept as pets can efficiently transmit zoonotic disease to humans. In addition, these animals may represent a niche for *Sarcoptes scabiei* infestation in countries where sarcoptic mange in commercial pig farms has been eradicated and could therefore pose, a hazard for specific pathogen free farms.

## Background

This case report describes a severe infestation of scabies in two miniature pigs kept as pets and subsequent zoonotic infection of a child.

Sarcoptic mange is regarded as one of the most important ectoparasitic diseases of swine worldwide, caused by the burrowing mite *Sarcoptes (S.) scabiei var. suis*, which shows a certain degree of host specificity [1–3]. However, it is a highly contagious skin disease with the potential to affect a variety of different animal host species [4]. All life stages of the parasite are found in either burrows of the epidermis or on the surface of the skin [1, 5]. Transmission mainly occurs directly, by prolonged skin to skin contact, but also indirectly, as mites may survive in humid and cold environment for several weeks [1, 6]. Mites can be found distributed over the entire body of infested hosts, but are generally concentrated in the ears and on the outer pinnae [2]. In pigs, two distinct clinical presentations of scabies are recognized and the occurrence of one or the other depends on the age of the animal. In growing pigs, a pruritic hypersensitive form is commonly seen, whereas a chronic, hyperkeratotic form with the presence of aural crusts and a large number of mites on the animal is recognized in sows [7]. A high prevalence of up to 95% within infected herds causes tremendous economic losses in swine reproduction and compromises animal welfare [2, 8]. A conclusive diagnosis of swine scabies is difficult, because a single, reliable and sensitive diagnostic tool is not available [9, 10]. The diagnosis in pigs with crusted scabies can usually be made easily, as thousands of mites are harbored in the skin and can be detected by skin scrapings. However, a timely diagnosis and successful elimination of mange can be challenging in pigs with only mild symptoms, due to the presence of only very few mites in the early stages of disease, leading to negative skin scrapings and asymptomatic or only mildly symptomatic animals ([1, 2, 7]. Furthermore, sarcoptic mange mimics other skin diseases such as atopic dermatitis, insect bites and skin conditions caused by irritating agents [1]. Thus, diagnostic blood tests for scabies have been developed to improve specificity and sensitivity [1]. Several ELISA systems are available to detect antibodies in infected pigs 5 to 6 weeks post infection with scabies mites [11]. In addition, examination of pig carcasses in the slaughterhouse provides further information on the scabies status of particular pig herds. In conclusion, clinical signs consistent with sarcoptic mange, microscopic analysis of skin scrapings and blood sampling for detection of specific antibodies by ELISA should ideally be combined to reach an accurate diagnosis.

Nowadays, miniature pigs have become popular companion animals and are more frequently seen by veterinarians [12]. While information on swine sarcoptic mange caused by *S. scabiei var. suis* is available, scabies in miniature pigs is rarely described in the scientific literature. “Dippity” syndrome is an important differential diagnosis in miniature pigs with skin lesions and behavioral problems. It is a poorly understood disease and once affected the animals recover within a few days without any treatment [13, 14].

Zoonotic transfer from infected pigs kept as pets has not been reported previously and little is known about zoonotic transmission of *Sarcoptes* mites from pigs to humans [15]. Transmission of the mite from infected domestic animals to humans occurs during close contact and causes intense pruritus and irritation in affected humans due to a hypersensitivity reaction against the mites and their products. Young children and immunocompromised adults are more susceptible to the disease [10]. Infestations are typically self-limiting but cases of persistent infection requiring several months to resolve have been described in the literature [16, 17].

## Case presentation

A 7- month-old, castrated male miniature pig with a body weight of 3.5 kg was referred for abnormal behavior consisting of continuous screaming, increased periods of lateral recumbency with uncontrolled pedaling motions, decreased activity and ataxia. The first symptoms appeared approximately 1 week prior to referral to the swine clinic. Previous therapy consisted of danofloxacin and corticosteroid injections with unknown dosage.

According to the owner, the other miniature pig, a castrated male littermate, did not show any abnormal clinical signs. Both animals shared an indoor pen in the house and an outdoor enclosure with a hut. The animals were fed a commercial horse feed combined with fresh vegetables and fruit, and offered water ad libitum. The miniature pigs had not been vaccinated and had never been treated for endo- or ectoparasites. Both animals had been bought 1 month before from a specialized miniature pig breeder. At presentation, the referred miniature pig was lethargic, and had a cachectic body condition. General examination revealed a slightly decreased body temperature of 36.5 °C (physiological range: 37–38 °C), a mildly increased heart rate of 100 beats per minute (physiological range: 68–98 per minute), and an increased respiratory rate of 36 (physiological range: 11–29 per minute).

**Fig. 1:**
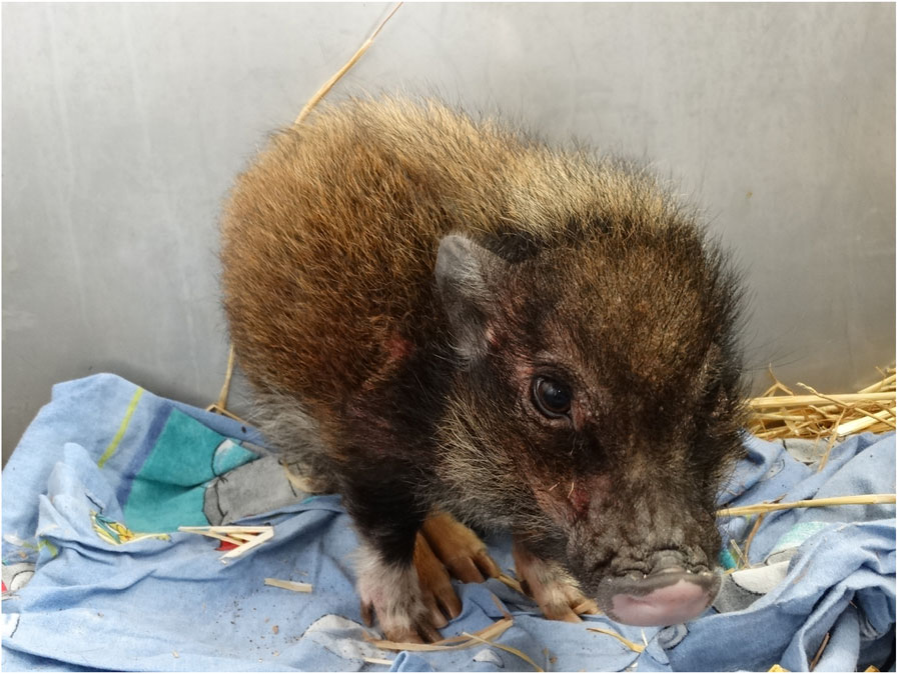
Clinical photograph of the severely affected miniature pig with generalized hypotrichosis, multifocal hyperkeratotic crusts and erythema

**Fig. 2:**
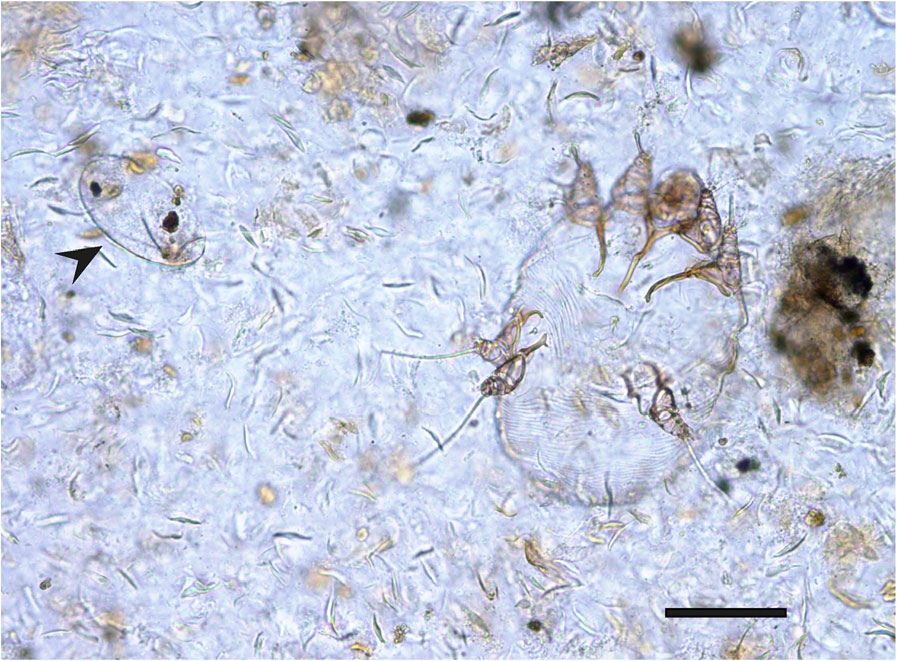
Photomicrograh of a Sarcoptes scabiei mite and an egg (arrow) in skin scrapings. Bar = 0.15 mm

Dermatological examination of the miniature pig revealed a dull, greasy coat with generalized hypotrichosis and multifocal erythema (Fig. 1). Extensive areas of hyperkeratosis were observed on the head, bilateral on the shoulders, extremities, the abdomen and perineal area. The entrance of both ear canals was obstructed with cerumen and squames, bordered by hyperkeratotic crusts. Microscopic examination of skin scrapings and cerumen revealed numerous *Sarcoptes* mites, instars, fecal pellets and eggs (Fig. 2). Cytological examination of the cerumen and crusts from the entrance to both ear canals revealed heavy colonization with rod-shaped bacteria, and a low number of coccoid bacteria and yeasts. Impression smears of the skin showed some neutrophils, extracellular cocci and corneocytes.

**Fig. 3:**
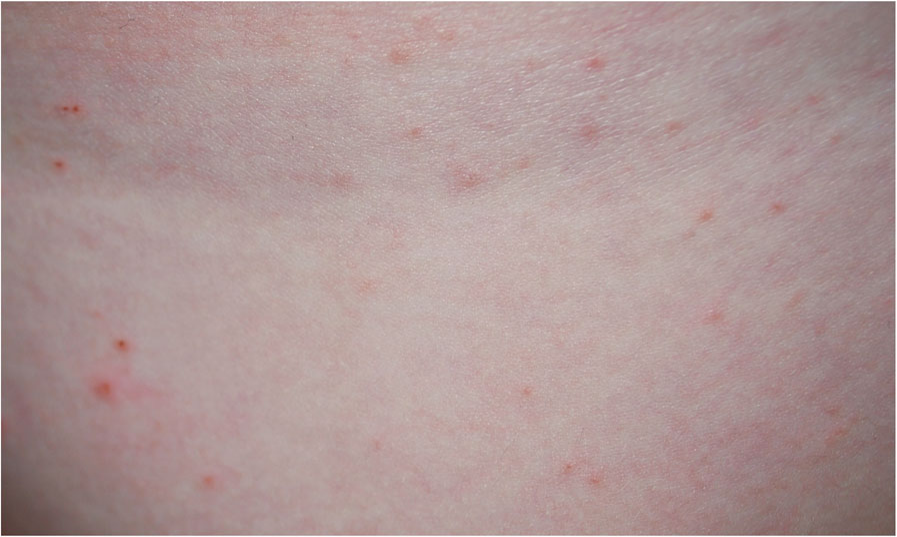
Papules and erythema on the leg of the infested child

Upon diagnosis of sarcoptic mange in this patient, the other miniature pig was also presented and examined. This animal showed a better body condition and comparable but milder skin lesions, consisting of mild hypotrichosis over the head and trunk and greasy skin with large scales. Pale hyperkeratotic crusts were absent. Microscopic examination revealed sarcoptes mites, but in smaller numbers. Further, the owner reported that her 7- year old daughter - often in contact with the miniature pigs - had recently developed pruritic papular skin lesions on the upper legs (Fig. 3) and was referred to a physician who confirmed a scabies infestation.

Both miniature pigs remained hospitalized for 14 days. During this time, an adequate feeding regime was started and a heating source was installed to control body temperature. Both pigs were treated with a subcutaneous injection of ivermectin (0.3 mg/kg; Ivomec®; Merial Ltd, Duluth, GA, USA), which was repeated after 2 weeks, combined with an intramuscular injection of butafosfan and cyanocobalamin (vitamin B12) (0.3 ml/kg; Catosal® 10%; Bayer, Shawnee Mission, KS, USA). Furthermore, the severely affected miniature pig was washed with a 3% chlorhexidine shampoo (Pyoderm; Virbac®, SA, Carros Cedex 06511, France) three times a week until scales and crusts resolved. The other miniature pig was washed only twice. No clinical adverse effects were observed throughout the treatment period. The owner was advised to remove the straw from the hut and to thoroughly clean both pens with hot water. After cleaning the outdoor area, the pen and hut were sprayed with a 1% cypermethrin solution (Intermitox®, SaVet Pharma GmbH, Vechta, Germany). The bedding of the indoor sleeping place was washed at 60 °C. The owner’s child was treated with an antipruritic ointment prescribed by the physician.

Skin scrapings were repeated after 2 weeks of hospitalization and were negative in both animals. Subsequently, both animals were discharged from the clinic. The owner was advised on how to feed the animals properly. Both miniature pigs were examined at their home at two and 4 weeks after their return. During this time, the animals further improved and displayed normal behavior and weight gain with complete remission of skin lesions. The owner was contacted by phone 4 months later and declared that both animals and the child remained without skin lesions.

## Discussion

Nowadays, miniature pigs are exotic but relatively popular pets [13]. In the last decades, the number of miniature pigs kept as pets increased significantly. However, finding veterinarians with expertise in this particular animal species may prove challenging for animal owners [14]. Hence, many miniature pigs do not receive the recommended preventive health care. Swine specialists have the best knowledge in treating pet pigs, but are not always within easy reach for the owners. Furthermore, owners can be easily offended if routine techniques to handle swine are used on their pets [14].

A survey of clinical problems identified in 102 miniature pigs revealed that the locomotor system and the integument were the most common locations for problems in pet pigs [18]. In our case, a *Sarcoptes* infestation was diagnosed in both miniature pigs. Depending on the country, sarcoptic mange can be either absent or endemic with variable prevalence in conventionally held pigs [19]. Although sarcoptic mange is eradicated in farmed pigs in Switzerland, infestations with *Sarcoptes* mites were recently recognised in wild boars [20]. However, the presented miniature pigs, not having any evidence for wild boar contact, had most likely been infected at the breeding farm. Based on history and on-site examination, any transmission by fomites or direct contact with wild boars or other pigs in their home environment was excluded.

Pruritus in artificially infested pigs may develop at the earliest 2–3 weeks post infestation but often starts later [21, 22]. Although intense pruritus is considered the hallmark sign of sarcoptes infestations in dogs and other species, it was not obvious as such in either presented miniature pigs. Neither of the animals was observed to scratch or rub themselves intensely during hospitalisation, but the pedalling motions in the recumbent animal may have been a sign of pruritus combined with general weakness. However, this animal ate with great appetite when offered food, and was much more active 1 day after the first treatment. Malnutrition caused by inappropriate diet may have contributed to the development of crusted scabies and atypical clinical signs in this miniature pig. Development of crusted scabies with a high mite burden is generally associated with an inadequate immune response and is often associated with immunosuppression in humans and dogs [23]. This observation is supported by the fact that *Sarcoptes*-infected pigs develop crusted scabies when treated with dexamethasone [24]. It remains a moot point whether the previous glucocorticoid injection contributed to the hyperkeratotic lesions in one of the miniature pigs.

Although the health status of adult pigs with crusted scabies has not been described in detail, it has not been reported either that these animals suffer from poor health. However, these animals typically do not show pruritus and therefore do not remove mites by pruritic behavior. This lack of pruritic behavior may have resulted in the large mite numbers found in the miniature pig described here.

Whereas the distribution of clinical lesions in pigs affected by sarcoptic mange usually includes the head and lateral side of the extremities, the proximal parts of the pinnae around the entrance to the ear canals were severely affected in one of the miniature pigs described here. Of note, this area is reported to be a predilection site with high mite burden [21, 25].

Thorough cleaning of the pigs’ enclosures is mandatory, as transmission of sarcoptes mites by fomites has been demonstrated in pigs [22]. Transmission of sarcoptes mites from dogs or other species to people and induction of pruritus in affected humans is well known. Close contact with infected animals and lack of host specificity are contributing factors [23]. More recently, DNA-based studies on sarcoptes mites from different animal hosts including humans indicate that there is only limited genetic diversity, and one clade included both human and pig genotypes [26]. This finding supports the likelihood of cross-species infestations. In the case described here, the clinical signs of pruritus and erythematous papules on the legs of the child, the time point of their appearance and later recovery were suggestive of infestation with *Sarcoptes scabiei var. suis.*

Sarcoptic mange in pigs is successfully managed by two subcutaneous injections of ivermectin or a single intramuscular injection of doramectin [27, 28]. Because of malnutrition and poor to moderate body condition, both miniature pigs described here received an injection with a metabolic stimulant containing butafosfan and cyanocobalamin [29, 30]. The additional chlorhexidine shampoo therapy was administered to counteract the changes in the skin microbiota as observed by cytology. Alterations in the skin microbiome were demonstrated in a porcine scabies model where an increase of *Staphylococcus* species and a shift within the staphylococcal population in pigs with scabies infestation were shown [31]. In principle, this could have implications for the human skin microbiota as well, especially when pigs are kept as companion animals in close proximity with their owners sharing the same environment [32].

## Conclusions

This report describes the first case of a severe infestation of scabies in two miniature pigs kept as pets causing zoonotic infection of a child.

Pruritus is a clinical hallmark of sarcoptes infestations in most species, but can be absent or aberrant in miniature pigs. The inadequate nutrition may have influenced the course of disease in the case presented. Keeping miniature pigs as pets facilitates zoonotic transfer of *Sarcoptes* mites and microbiota. In addition, these animals may represent a niche for *Sarcoptes scabiei* infestation in countries where sarcoptic mange in commercial pig farms has been eradicated and can therefore pose a hazard for specific pathogen free farms.
